# Distribution of *Candida* species isolated from people living with human immunodeficiency virus with oropharyngeal and oral candidiasis in Africa in the era of universal test and treat policy: a systematic review and meta-analysis

**DOI:** 10.1186/s41182-024-00649-6

**Published:** 2024-11-27

**Authors:** Benson Musinguzi, Ekwaro A. Obuku, Alex Mwesigwa, Richard Migisha, Alison Annet Kinengyere, Regina Ndagire, Andrew Baguma, Erick Jacob Okek, Ronald Olum, Herbert Itabangi, Gerald Mboowa, Obondo James Sande, Beatrice Achan

**Affiliations:** 1 Department of Immunology and Molecular Biology, School of Biomedical Sciences, College of Health Sciences, Makerere University, Kampala, Uganda; 2 Department of Medical Laboratory Science, Faculty of Health Sciences, Muni University, Arua, Uganda; 3 Africa Centre for Systematic Reviews and Knowledge Translation, College of Health Sciences, Makerere University, Kampala, Uganda; 4 Clinical Epidemiology Unit, School of Medicine, College of Health Sciences, Makerere University, Kampala, Uganda; 5 Faculty of Epidemiology and Population Health, London School of Hygiene & Tropical Medicine, University of London, London, UK; 6 Department of Global Health Security, Infectious Diseases Institute, College of Health Sciences, Makerere University, Kampala, Uganda; 7 Department of Microbiology, School of Medicine, Kabale University, Kabale, Uganda; 8 Department of Physiology, Mbarara University and Science and Technology, Mbarara, Uganda; 9 Sir Albert Cook Medical Library, College of Health Sciences, Makerere University, Kampala, Uganda; 10 School of Medicine, College of Health Sciences, Makerere University, P.O. Box 7062, Kampala, Uganda; 11 Department of Microbiology and Immunology, Faculty of Health Sciences, Busitema University, Mbale, Uganda; 12 African Centre of Excellence in Bioinformatics and Data Intensive, Sciences, the Infectious Diseases Institute, College of Health Sciences, Makerere University, Kampala, Uganda; 13 Department of Medical Microbiology, School of Biomedical Sciences, College of Health Sciences, Makerere University, P.O. Box 7072, Kampala, Uganda

**Keywords:** Candida, *C. albicans*, Oropharyngeal, Oral, Candidiasis, Test and treat

## Abstract

**Background:**

The introduction of antiretroviral therapy (ART) and the implementation of the human immunodeficiency virus (HIV) universal test and treat (UTT) policy have led to a decline in the incidence of opportunistic infections. However, oropharyngeal and oral candidiasis remain prevalent and continue to pose challenges among people living with human immunodeficiency virus (PLHIV) in Africa, indicating the need for a better understanding of the distribution of *Candida* species responsible for these infections. This systematic review and meta-analysis aimed to determine the distribution of *Candida* species isolated from PLHIV with oropharyngeal and oral candidiasis in Africa in the era of UTT policy**.**

**Methods:**

The review followed the preferred reporting items for systematic review and meta-analysis (PRISMA) guidelines. A comprehensive search was conducted to identify eligible studies to be included in the meta-analysis and analysed using a random effects model in STATA version 17.

The risk of bias was assessed using the Joanna Briggs Institute quality assessment tool.

**Results:**

Fourteen studies with 4281 participants were included in the review. Overall, 2095 *Candida* isolates were reported, 78.7% (1650/2095) of which were *C. albicans,* 19.6% (410/2095)*,* non-*albicans Candida* (NAC), and 1.7% (35/2095) could not be identified to the *Candida* specific species level. The most prevalent NAC species were *C. glabrata* (26.3%), followed by *C. tropicalis* (24.9%), *C. krusei* (15.6%), *C. parapsilosis* (11%), and *C. dubliniensis* (6.3%). The pooled prevalence of oropharyngeal and oral candidiasis was 48% (95% CI 34–62%). The prevalence of oropharyngeal candidiasis was higher in the pre-UTT era, at 56% (95% CI 40–72%, *p* < 0.001), than in the post-UTT era, at 34% (95% CI 10–67%, *p* < 0.001). The risk of bias assessment revealed that 71.4% (10/14) of the included studies had a low risk of bias and that 28.6% (4/14) had a moderate risk of bias.

**Conclusions:**

While *C. albicans* remain, the predominant species causing oropharyngeal and oral candidiasis among PLHIV in Africa, NAC species also contribute significantly to the infection burden. Despite ART and UTT policies, oropharyngeal candidiasis remains prevalent, emphasizing the need for targeted interventions.

## Background

As of 2022, approximately 38 million people were living with human immunodeficiency virus (HIV) globally, with approximately 68% (25.7 million) of these individuals residing in Africa [1]. Oropharyngeal candidiasis and oral candidiasis are opportunistic mucosal fungal infections that commonly affect the oral mucosa of the oral cavity, with oropharyngeal candidiasis extending to affect the throat and invading the epithelial cell lining of the oropharynx [2]. Oropharyngeal candidiasis and oral candidiasis are considered important indicators of immune suppression and progression to acquired immunodeficiency syndrome (AIDS) in people living with human immunodeficiency virus (PLHIV) [2]. Oropharyngeal candidiasis occurs in approximately 90% of PLHIV when CD4 T-cell counts drop below 200 cells/μL [3–5]. The prevalence varies globally, from 17.8 to 44.2% in India [6–8], 66.7% in Brazil [9], 31.6% in Mexico [10], and 4.9% to 79.4% in African countries [11, 12]. In Africa, where HIV prevalence is high and healthcare resources are limited, the burden of oropharyngeal and oral candidiasis among PLHIV is a concern, as severe forms can lead to swallowing difficulties, reduced food intake, oral cancer, and impaired quality of life [13].

While *C. albicans* remains the most common cause of oropharyngeal and oral candidiasis, accounting for 48% to 87% of cases [14], there has been a reported shift towards non-*albicans Candida* (NAC) species [15, 16], such as *C. parapsilosis, C. glabrata, C. tropicalis, C. dubliniensis*, *C. krusei* and *C. guilliermondii* [17]. Furthermore, multidrug-resistant *Candida auris* strains are emerging as significant nosocomial pathogens worldwide [18, 19].

The diagnosis of oropharyngeal candidiasis and oral candidiasis is often based on clinical presentation without identifying the causative agent; however, empirical management is often inadequate, and suboptimal management can lead to antifungal resistance, persistent symptoms, and life-threatening dissemination, significantly impacting the quality of life of PLHIV [13].

Over the years, key interventions to improve the health outcomes of PLHIV have been explored [20, 21]. In the early 2000s, advancements in antiretroviral therapy (ART) increased accessibility for HIV patients on the basis of the CD4 cell count or clinical stage [21]. In 2015, the WHO introduced the universal test and treat (UTT) policy, which recommends immediate ART initiation for all individuals diagnosed with HIV, regardless of their CD4 count or clinical stage [20]. This policy has significantly increased the number of PLHIV on ART, leading to improved immune function, reduced HIV transmission rates, and a decreased incidence of opportunistic infections [22]. However, oropharyngeal candidiasis and oral candidiasis continue to pose challenges among PLHIV in resource-limited African countries or those with deprived immunologic responses [23, 24]. The impact of the UTT policy on the distribution of *Candida* species isolated from PLHIV with oropharyngeal and oral candidiasis in African countries remains unclear.

In addition, oropharyngeal candidiasis causes discomfort, pain, difficulty swallowing, and altered taste sensation [23], which can also make it difficult for PLHIV to adhere to their ART regimen, increasing the risk of HIV drug resistance and developing oral noncommunicable diseases (NCDs) such as oral cancer [23]. Oropharyngeal candidiasis in PLHIV not only has psychosocial impacts, such as stigma and discrimination but also has economic consequences, increasing healthcare costs and potentially leading to reduced productivity.

Understanding the distribution of *Candida* species isolated from PLHIV with oropharyngeal and oral candidiasis in Africa before and after the UTT policy is essential for the diagnosis and management of oropharyngeal candidiasis and oral candidiasis. In addition, it is crucial for designing effective HIV care programs that address comorbidities, prevent drug resistance and enhance the overall health outcomes and well-being of PLHIV. Thus, this systematic review and meta-analysis aimed to determine the distribution of *Candida* species isolated from PLHIV with oropharyngeal and oral candidiasis in Africa in the era of UTT policy.

## Materials and methods

### Study design

This systematic review and meta-analysis were conducted according to the preferred reporting items for systematic review and meta-analysis (PRISMA) guidelines [25]. The protocol of this review was registered in the open access PROSPERO database before the review was conducted, number CRD42021254473 (https://www.crd.york.ac.uk/prospero/).

### Data sources

With the assistance of an experienced librarian and information scientist, searches were conducted in the PubMed (https://pubmed.ncbi.nlm.nih.gov/), Scopus (https://www.Scopus.com/home.uri), and EMBASE (https://www.embase.com) databases for relevant English-language articles. In addition, the reference lists of all identified studies were searched for relevant articles, and gray literature was searched for in Google Scholar (https://scholar.google.com/). The search was restricted to the period from January 1, 2000, to July 1, 2024. All the articles were exported to Mendeley Reference Manager v2.120.0 software (Mendeley Ltd., London, UK) for further processing, and duplicates were removed.

### Search strategy

The search terms were combined using Boolean operators OR for synonyms and ‘AND’ across elements of PECO (population, exposure, comparator, outcome), and the study design was as follows:

The terms for the population of interest were ‘HIV’, ‘AIDS’, ‘human immunodeficiency virus’, and ‘acquired immune deficiency syndrome’. This population was restricted to sub-Saharan Africa by country name: Africa OR Algeria OR Angola OR Benin OR Botswana OR Burkina Faso OR Burundi OR Cameroon OR Canary Islands OR Cape Verde OR Central African Republic OR Chad OR Comoros OR Democratic Republic of Congo OR Djibouti OR Egypt OR Equatorial Guinea OR Eritrea OR Ethiopia OR Gabon OR Gambia OR Ghana OR Guinea OR Guinea OR Guinea Bissau OR Ivory Coast OR Kenya OR Lesotho OR Liberia OR Libya OR Libi OR Libia OR Madagascar OR Malawi OR Mali OR Mauritania OR Mauritius OR Morocco OR Mozambique OR Mocambique OR Namibia OR Niger OR Nigeria OR Principe OR Reunion OR Rwanda OR Sao Tome OR Senegal OR Seychelles OR Sierra Leone OR Somalia OR South Africa OR St Helena OR Sudan OR Swaziland OR Tanzania OR Togo OR Tunisia OR Uganda OR Western Sahara OR Zaire.

The search terms for exposure were ‘non-*albicans Candida’*, ‘NAC’, ‘Candida*’,* ‘*C. albicans’*, ‘*C. parapsilosis’,* ‘*C. glabrata’, ‘C. tropicalis’, ‘C. dubliniensis’*, *C. krusei’*, ‘*C. norvegensis’,* ‘ *C. guilliermondii’, C. albicans’,* ‘*C. glabrata’,* ‘*C. tropicalis’,* ‘*C. krusei’,* ‘*C. dubliniensis’,* ‘*C. parapsilosis’,* ‘*C. guilliermondii’,* ‘*C. famata’, ‘C. kefyr’,* ‘*C. norvegensis’,* ‘*C. sake’,* ‘*C. lusitaniae’,* ‘*C. pintolopesii’,* ‘*C. pseudotropicalis’,* ‘*C. globosa’,* ‘*C. dattila’,* ‘*C. inconspicua’,* ‘*C. hellenica’,* ‘*C. holmii’,* ‘*C. pulcherrima’,* ‘*C. valida’,* ‘*C. africana’,* ‘*C. fabianii’,* ‘*C. cacaoi’,* ‘*C. zeylanoides’.*

The search terms for comparator were ‘antiretroviral’, ‘therapy*’,* ‘universal*’,* ‘test*’,* and ‘treat*’.*

The search terms for the outcome of interest were ‘oropharyngeal’, ‘candidiasis’, ‘OPC’, ‘Oral’, and ‘thrush’.

The search terms for the study design were ‘cross-sectional*’,* ‘observational*’,* ‘descriptive*’,* ‘prevalence*’,* ‘transverse*’,* ‘cohort*’,* and ‘case‒control*’.*

This search was restricted to the period from January 1, 2000, to July 1, 2024. In addition, the reference lists of all included studies were searched on Google Scholar for more articles.

### Review question and eligibility criteria

The review question was “What is the distribution of *Candida* species isolated from PLHIV with oropharyngeal and oral candidiasis living within Africa?” As a quantitative systematic review, this question was described further (Table 1) using the PECOST framework, which guided the eligibility of the included studies. Studies were included if they were published in the English language between January 1, 2000, and July 1, 2024, and if they reported outcomes of interest, that is, the prevalence of oropharyngeal or oral candidiasis and distribution of *Candida* species among PLHIV in Africa. Studies that conducted data collection from January 1, 2000, to December 31, 2014, were classified as belonging to the pre-UTT. Conversely, studies that collected data between January 1, 2015, and July 1, 2024, were categorized as part of the UTT era. This review included only observational studies with either cross-sectional, case‒control or cohort designs reporting oropharyngeal candidiasis or oral candidiasis and *Candida* species among PLHIV living in Africa. We included studies that diagnosed oropharyngeal candidiasis or oral candidiasis infection on the basis of both the presence of oral lesions and the mycological identification of *Candida* species isolated from the oropharynx or/and oral cavity of PLHIV. We excluded studies that reported the clinical prevalence of oropharyngeal candidiasis or oral candidiasis without information on the causative *Candida* species. We excluded animal model reports and observational studies whose full text could not be retrieved even after request from the corresponding authors and a comprehensive library search.

**Table 1:** Description of the “PECOST” elements for the systematic review of oropharyngeal or oral candidiasis

Element	Description
Population	People living with HIV in Africa
Exposure	*Candida species*
Comparator	pre-UTT policy era and post-UTT policy era
Outcome	The primary outcome of this review was the distribution of *Candida* species isolated from of PLHIV with oropharyngeal and oral candidiasis in Africa
Study design	Observational study design (cross-sectional studies, cohort studies, and case‒control studies)
Timelines	January 1, 2000 to July 1, 2024

### Study outcomes

The primary study outcome was the distribution of *Candida* species isolated from PLHIV with oropharyngeal and oral candidiasis, and the prevalence of oropharyngeal and oral candidiasis in PLHIV was the secondary outcome (Table 1).

### Study selection process

#### Data management

**Fig. 1:**
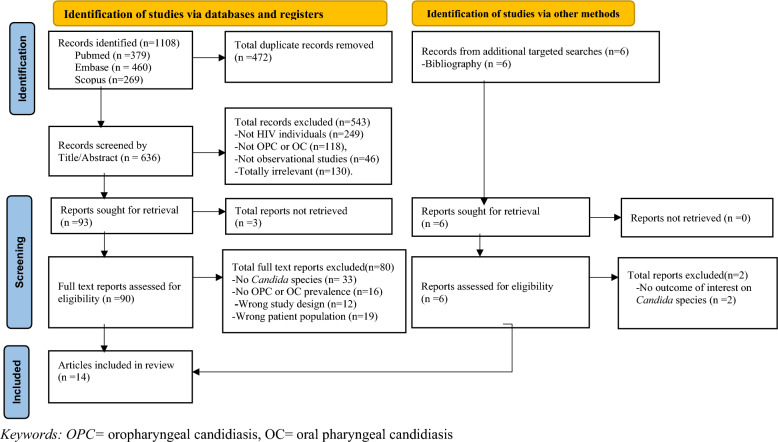
PRISMA flow chart showing the literature search and selection process

Using Mendeley Desktop referencing software version 1.19.8 (Mendeley Ltd., London, UK), we imported all identified titles, excluded duplicates, and screened and grouped these into relevant eligibility categories as described in our PRISMA flow chart (Fig. 1).

#### Minimizing *bias* in study identification and selection

Two reviewers (BM and AAK) carefully conducted the literature search. Two independent reviewers (HI and GM) examined relevant studies and screened their titles and abstracts for eligibility. After the initial screening, the full texts of the eligible studies were retrieved and examined for eligibility by RN and AM. Disagreements were resolved by discussion with two reviewers (BA and OJS) to reach a consensus.

#### Data extraction

Data extraction was performed using a spreadsheet developed from Microsoft Excel version 16 (Microsoft Corporation, Richmond, Seattle, Washington, USA). The extracted data included the first author, year of publication, country where the study was conducted, African region where the study was conducted, year of data collection, UTT era, sample size, sex, mean age of the study population, *Candida* identification method, and prevalence of oropharyngeal candidiasis or oral candidiasis and *Candida* species. The data were extracted in duplicate by RO and RN, and any disagreements were resolved by a third party (BM).

#### Operational definitions

We included only studies that identified *candida* species in PLHIV with either oropharyngeal or oral candidiasis with a stated prevalence of infection.

The pre-UTT period was defined as the period (2000–2014) when ART was made available and initiation was based on the CD4 count and WHO clinical stage, whereas the post-UTT period was defined as the period since the WHO rolled out the UTT policy in 2015 to date, which included PLHIV regardless of the HIV clinical stage, and the CD4 cell count was initiated on lifelong ART. All the studies whose data were collected between 2000 and 2014 were considered pre-UTT, whereas those whose data were collected between 2015 and 2024 were considered post-UTT.

During trend analysis, we defined cumulative prevalence as the proportion of PLHIV with oropharyngeal or oral candidiasis that occurred from January 1, 2000, to July 1, 2024.

#### Qualitative assessment

Two reviewers (GM and BM) independently assessed the risk of bias in the included studies, and any discrepancies between the two reviewers were resolved by reaching a consensus through discussion. Eligible studies were assessed for risk of bias using the Joanna Briggs Institute quality assessment tool for prevalence studies [26]. This tool consists of 9 parameters: (1) an appropriate sampling frame to address the target population, (2) a proper sampling method, (3) an adequate sample size, (4) a description of the study subject and setting, (5) sufficient data analysis, (6) the use of valid methods for the identified conditions, (7) valid measurements for all participants, (8) the use of appropriate statistical analysis, and (9) an adequate response rate. Each criterion was scored as 1 for failure to meet the requirement or 0 for meeting the requirement. The overall risk of bias was categorized as low (score 5–9), moderate (score 3–4), or high (score 0–2) (Table 4).

#### Data analysis

The extracted data were cleaned and imported into STATA 17.0 statistical software (STATA, College Station, Texas, USA) for analysis. Descriptive statistics and narrative synthesis were used to summarize the data and present the results. A random effect meta-analysis model was used to estimate the pooled prevalence of oropharyngeal and oral candidiasis as well as *Candida* species in Africa. Subgroup meta-analyses were performed by clinical condition (oropharyngeal candidiasis and oral candidiasis), region and UTT era.

Heterogeneity across studies was assessed using Q statistics and reported as *I*^*2*^. Egger’s test for small study effects and funnel plots were used to assess publication bias. Trim-and-fill methods were applied to correct possible publication bias. These results are displayed in a forest plot. Because we found a high level of heterogeneity, we conducted a meta-regression testing the variables of the year of data collection, year of publication, African region, and UTT era to rule out sources of publication bias. Any value with *p* < 0.05 was considered statistically significant at the 95% confidence interval (CI).

#### Meta-regression

Meta-regression analysis was performed to explore the associations between the prevalence of oropharyngeal/oral candidiasis and the year of data collection, year of publication, African region, and UTT era.

## Results

### Search results

The PRISMA flow chart summarizes the identified, screened, excluded, and included studies with reasons for exclusion. The database search yielded 1108 titles from the PubMed (*n* = 379), EMBASE (*n* = 460) and Scopus (*n* = 269) databases. After removing duplicates (*n* = 472), 636 titles and abstracts were screened, and 543 studies were excluded, mainly because of the absence of a PLHIV study population (*n* = 249), lack of oropharyngeal or oral candidiasis outcomes of interest (*n* = 118), non-observational study design (*n* = 46) and irrelevant studies (*n* = 130). A total of 93 studies were sought for retrieval, and 90 full-text records were successfully retrieved; however, 3 articles were not retrievable and were excluded. In the full-text screening, 10 articles were fit for inclusion in the review, and 80 studies were excluded because of a lack of information on the outcome of interest, an incorrect population and the study design. The reference lists of the included articles were searched for additional relevant articles, and 6 articles were retrieved and screened for eligibility. Two articles were excluded because of a lack of information on *Candida* species. Four studies were included for review. A total of 14 studies (10 from databases and 4 from additional targeted searches) were included in the review and meta-analysis (Fig. 1).

### Summary of included studies

Fourteen [14] observational studies reporting the distribution of *Candida* species and the prevalence of oropharyngeal candidiasis among PLHIV in 7 countries were fully reviewed and included in the meta-analysis. Among 14 studies, 11 reported both the distribution of *Candida* species and the prevalence of oropharyngeal candidiasis, whereas 3 studies reported the distribution of *Candida* species and the prevalence of oral candidiasis. These studies were conducted in Nigeria (*n* = 4) [27–30], Cameroon (*n* = 3) [16, 31, 32], South Africa (*n* = 2) [31, 33], Uganda (*n* = 2) [15, 34], Ghana (*n* = 1) [35], Chad (*n* = 1) [14] and the Ivory coast (*n* = 1) [12] (Table 2).

**Table 2:** Summary of included studies on oropharyngeal candidiasis and oral candidiasis in PLHIV in different African countries

No	Author, Year of Publication	Country	Region	Sample size	Gender M F		Age in years (Range/Mean)	Clinical condition	Year of data collection	UTT era	Candida Identification Method	OPC Prevalence n (%)	Total Candida isolates (n)	95% CI	
														Lower limit	Upper limit
1	Agwu et al. 2012 [34]	Uganda	East Africa	605	136	469	6–75	OPC	2011	Pre-UTT	Chromo agar, API32, PCR	315 (52)	315	0.48	0.56
2	Nweze & Ogbonnaya, 2011 [28]	Nigeria	West Africa	200	^a^	^a^	^a^	OPC	2009	Pre-UTT	Chromo agar, Api29x	120 (60)	120	0.53	0.67
3	Osaigbovo et al. 2017 [11]	Nigeria	West Africa	350	88	262	18–75 (41.6)	OPC	2017	Post-UTT	CHROMagar, germ tube test, API Candida	17 (4.9)	`	0.026	0.071
4	Enwuru et al. 2008 [27]	Nigeria	West Africa	213	105	108	≥ 18	OPC	2005	Pre-UTT	Germ tube, Sugar Fermentation	68 (31.9)	68	0.26	0.38
5	Kwamin et al. 2013[35]	Ghana	West Africa	267	98	169	15–74	OPC	2009	Pre-UTT	API ID32C	201 (75.3)	201	0.70	0.80
6	Ambe et al. 2020 [16]	Cameroon	Central Africa	378	102	276	3–72 (40.3)	OPC	2018	Post-UTT	Germ tube test, Chromo agar	162 (43)	171	0.38	0.48
7	Miguel et al. 2013a [31]	Cameroon	Central Africa	262	^a^	^a^	^a^	OPC	2012	Pre-UTT	Chromo agar, germ tube	126 (48|)	126	0.42	0.54
8	Taverne-Ghadwal et al. 2022 [14]	Chad	Central Africa	247	185	62	2–70 (34)	OC	2021	Post-UTT	MALDI–TOF MS, Rice, and Staib agar, API, PCR	119 (48.2)	129	0.42	0.54
9	Owotade & Patel, 2014 [33]	South Africa	Southern Africa	197	0	197	38.3	OC	2013	Pre-UTT	chromo agar, api20x	15 (7.6)	15	0.04	0.11
10	Konaté et al. 2017[12]	Ivory Coast	West Africa	286	^a^	^a^	15–63 (39.2)	OPC	2011	Post-UTT	germ tube, API 20	227 (79.4)	227	0.75	0.84
11	Miguel et al. 2013b [31]	South Africa	Southern Africa	212	^a^	^a^	^a^	OPC	2012	Pre-UTT	chromo agar, germ tube	168 (79)	128	0.74	0.85
12	Ekwealor et al. 2023 [30]	Nigeria	West Africa	150	53	97	< 20 to > 40	OC	2023	Post-UTT	chromo agar, PCR	98 (65.3)	98	0.58	0.73
13	Musinguzi et al. 2024 [15]	Uganda	East Africa	384	93	291	43.5	OPC	2023	Post-UTT	MALDI–TOF MS,	29 (7.6)	35	005	0.10
14	Yongabi et al. 2009 [32]	Cameroon	Central Africa	530	212	318	^a^	OPC	2008	Pre-UTT	Germ tube, Sugar Fermentation	387 (73)	387	0.69	0.77

*OPC* oropharyngeal candidiasis, *OC* oral candidiasis, *UTT* universal test and treatment, *API *analytical profile index, *CI* confidence interval, *PCR* polymerase chain reaction, *ID* identification, *MALDI–TOF MS* matrix-assisted laser desorption/ionization–time-of-flight mass spectrometry

^a^information not provided

All 14 studies had a total sample size of 4281 participants. The largest study had a sample size of 605 participants, while the smallest study had 150 participants (Table 2).

### Findings on the outcomes of interest

#### Distribution of *Candida* species isolated from PLHIV with oropharyngeal candidiasis or oral candidiasis

A total of 2095 *Candida* isolates were reported in 7 African countries, with approximately 32.6% (684/2095) from Cameroon [16, 31, 32], 17.2% (361/2095) from Nigeria [27–30],16.7% (350/2095) from Uganda [15, 34], 10.8% (227/2095) from the Ivory coast, 9.6% (201/2095) from Ghana [35], 6.2% (129/2095) from Chad [14] and 6.8% (143/2095) from South Africa [31, 33] (Table 3). Approximately 78.7% (1650/2,095) were *C. albicans,* 19.6% (410/2,095) were NAC isolates, and 1.7% (35/2,095) of the isolates were not identified at the *Candida* species level.

**Table 3:** Distribution of Candida species isolated from people living with HIV across different studies and countries

Study	Country	*Number of isolates (N)*	*Candida *species n (%)											
			*C. albicans*	NAC species										
				*C. glabrata*	*C.tropicalis*	*C.krusei*	*C.dubliniensis*	*C.parapsilosis*	*C.guilliermondii*	*C.famata*	*C. kefyr*	*C.norvegensis*	*C. sake*	*C. lusitaniae*
Agwu et al. 2012 [34]	Uganda	315	274 (87)	5 (1.6)	5 (1.6)			2 (0.6)				4 (1.3)	1 (0.3)	
Nweze & Ogbonnaya, 2011 [28]	Nigeria	120	54 (45)		22 (18.3)	2 (1.7)	9 (7.5)	18 (15)	11 (9.2)		2 (1.7)			2 (1.7)
Osaigbovo et al. 2017 [11]	Nigeria	75	61 (81.3)	4 (5.3)	2 (2.7)	2 (2.7)		5 (6.7)		1 (1.3)				
Enwuru et al. 2008 [27]	Nigeria	68	30 (44.1)	4(5.9)	13 (19.1)	5 (7.4)	1 (1.5)	3 (4.4)	1 (1.5)	3 (4.4)	2 (2.9)			
Kwamin et al. 2013[35]	Ghana	201	139 (69.2)	2 (1)	15 (7.5)	13 (6.5)	3 (1.5)	6 (3)	2 (1)	2 (1)	1 (0.5)	2 (1)	5 (2.5)	2 (1)
Ambe et al. 2020 [16]	Cameroon	171	103 (60.2)	29 (16.9)	11 (6.4)	21 (12.3)		4 (2.3)						
Miguel et al. 2013a [31]	Cameroon	126	92 (73)	24 (19)	4 (3.2)	3 (2.4)	1 (0.8)							
Taverne-Ghadwal et al. 2022 [14]	Chad	129	95 (73.6)	3 (2.3)	8 (6.2)	9 (6.9)		4 (3.1)	1 (0.8)		1 (0.8)			
Owotade and Patel, 2014). [33]	South Africa	15	12 (80)		1 (1.7)					2 (13.3)				
Konaté et al. 2017 [12]	Ivory coast	227	216 (95.2)	3 (1.3)	5 (2.2)			1 (0.4)						
Miguel et al. 2013b [31]	South Africa	128	106 (82.8)	12 (9.4)			10 (7.8)							
Ekwealor et al. 2023 [30]	Nigeria	98	61 (62.2)	18 (18.4),	12 (12.2)	7 (7.2)								
Musinguzi et al. 2024 [15]	Uganda	35	20 (57.1)	4 (11.4)	4 (11.4)	2 (5.7)	2 (5.7)	2 (5.7)						1 (2.9)
Yongabi et al. 2009 [32]	Cameroon	387	387 (100)											
Total		2095	1650	108	102	64	26	45	15	8	6	6	6	5

Study	Coun try	Number of isolates (N)	*Candida *species n (%)										Unidentified	
			NAC species											
			*C. pseudotropicalis*	*C. globosa*	*C. dattila*	*C. inconspicua*	*C. hellenica*	*C. holmii*	*C. pulcherrima*	*C. valida*	*C. fabianii*	*C. cacaoi*		
Agwu et al. 2012 [34]	Uganda	315											24 (7.6)	
Nweze & Ogbonnaya, 2011 [28]	Nigeria	120												
Osaigbovo et al. 2017 [11]	Nigeria	75												
Enwuru et al. 2008 [27]	Nigeria	68	3 (4.4)										3 (4.4)	
Kwamin et al. 2013[35]	Ghana	201		3 (1.5)	1 (0.5)	1 (0.5)	1 (0.5)	1 (0.5)	1 (0.5)	1 (0.5)				
Ambe et al. 2020 [16]	Cameroon	171	3 (1.8)											
Miguel et al. 2013a [31]	Cameroon	126											2 (1.6)	
Taverne-Ghadwal et al. 2022 [14]	Chad	129									1 (0.8)	1 (0.8)	6 (4.7)	
Owotade and Patel, 2014). [33]	South Africa	15												
Konaté et al. 2017 [12]	Ivory coast	227				2 (0.9)								
Miguel et al. 2013b [31]	South Africa	128												
Ekwealor et al. 2023 [30]	Nigeria	98												
Musinguzi et al. 2024 [15]	Uganda	35												
Yongabi et al. 2009 [32]	Cameroon	387												
Total		2095	6	3	1	3	1	1	1	1	1	1	35	

The prevalence of *C. albicans* ranged from 44.1% in Nigeria [27] to 100% in Cameroon [32] (Table 3). Regionally, in Central Africa, the prevalence of *Candida albicans* was 60.2%, 73% and 100% in 3 studies performed in Cameroon [16, 31, 32] and 73.6% in Chad [14]. In East Africa, the prevalence rates were 57.1% and 87% in Uganda [15, 34]. In Southern Africa, South Africa reported rates of 80% and 82.8% [31, 33]. In West Africa, *C. albicans* prevalence rates ranged from 44.1% to 81.3%, in Nigeria [27–30], 69.2% in Ghana [35] and 95.2% in the Ivory coast [12] (Table 3). Among the 410 NAC isolates, 26.3% (108/410) were *C. glabrata*, 24.9% (102/410) were *C. tropicalis*, 15.6% (64/410) were *C. krusei*, 11% (45/410) were *C. parapsilosis* and 6.3% (26/410) were *C. dubliniensis* (Table 3). Some rare NAC species were country-specific, such as *C. dattila*, *C. hellenica*, and *C. holmii* in Ghana, and *C. fabiani* and *C. cacaoi* in Chad. Uganda had the highest proportion of unidentified Candida species (68.6%, 24/35) (Table 3).

#### Overall pooled prevalence of *C. albicans* and NAC species isolated across studies

**Fig. 2:**
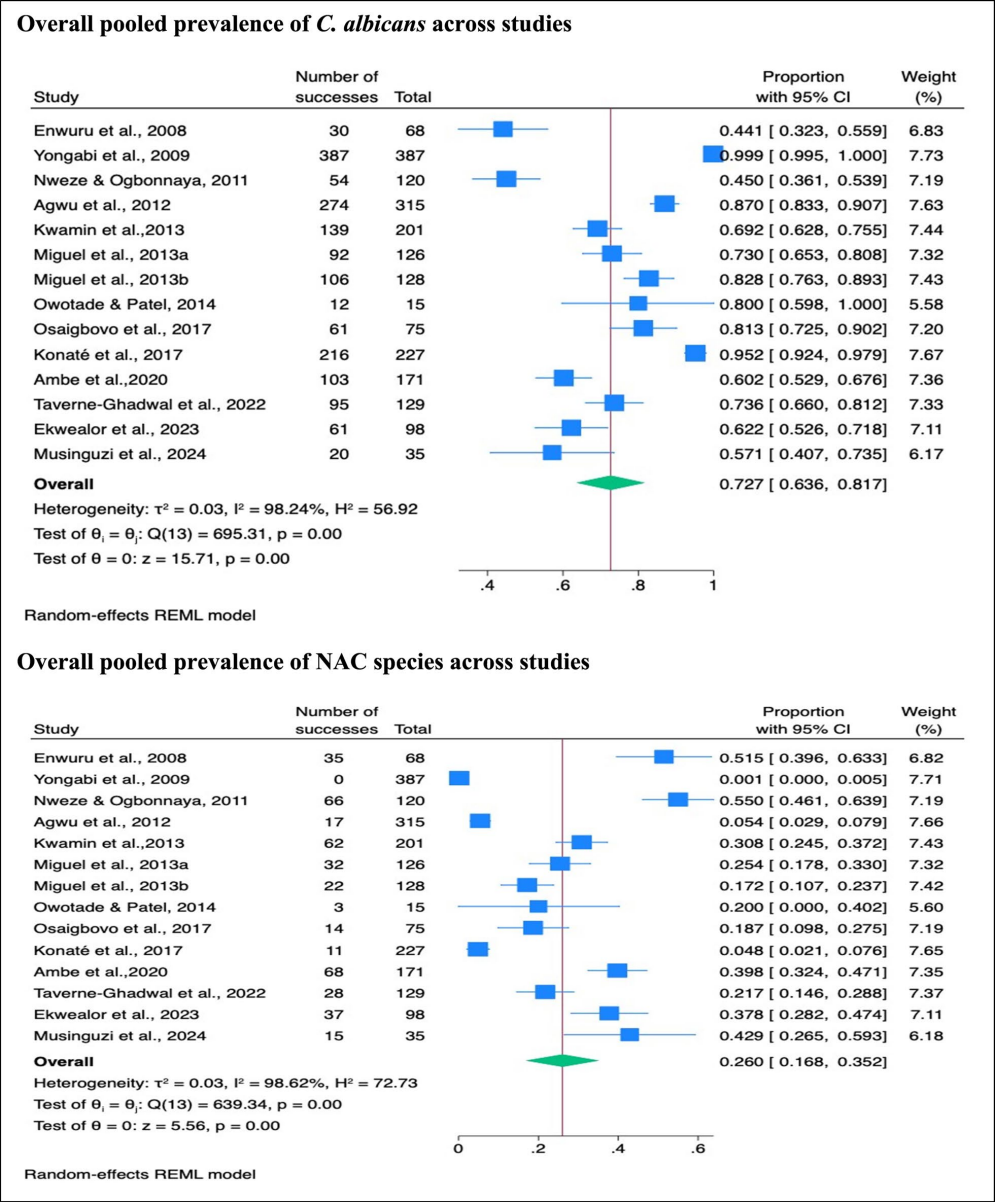
Pooled prevalence of *C. albicans* and NAC species across all studies

In the meta-analysis, the pooled prevalence of *C. albicans* was 73% (95% CI 64–82%, *p* < 001), and that of NAC species was 26% (95% CI 17–35%, *p* < 001) (Fig. 2).

#### Subgroup prevalence of *C. albicans* and NAC species in oropharyngeal and oral candidiasis

**Fig. 3:**
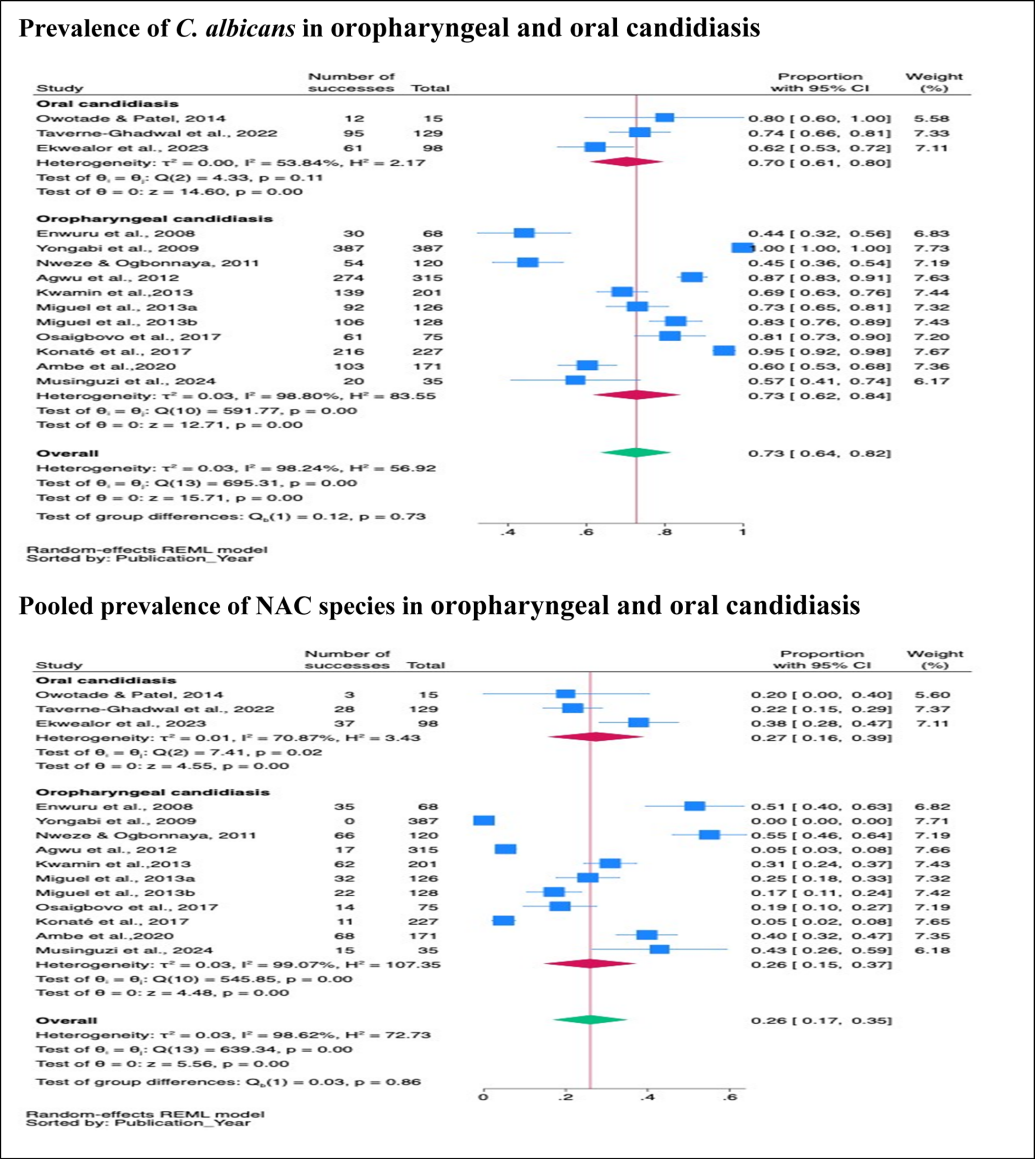
Pooled prevalence of *C. albicans* and NAC species in oropharyngeal and oral candidiasis

Subgroup analysis showed no significant difference in the prevalence of *C. albicans* between oropharyngeal candidiasis (73%, 95% CI 62–84%, *p* < 001) and oral candidiasis (70%, 95% CI 61–80%, *p* < 001), (*p* = 0.73). Similarly, the prevalence of NAC species showed no significant difference between oropharyngeal candidiasis (26%, 95% CI 15–37%, *p* < 001) and oral candidiasis (27%, 95% C: 16–39%, *p* < 001) (*p* = 0.80) (Fig. 3).

#### The pooled prevalence of *C. albicans* and NAC species during the pre- and post-UTT era

**Fig. 4:**
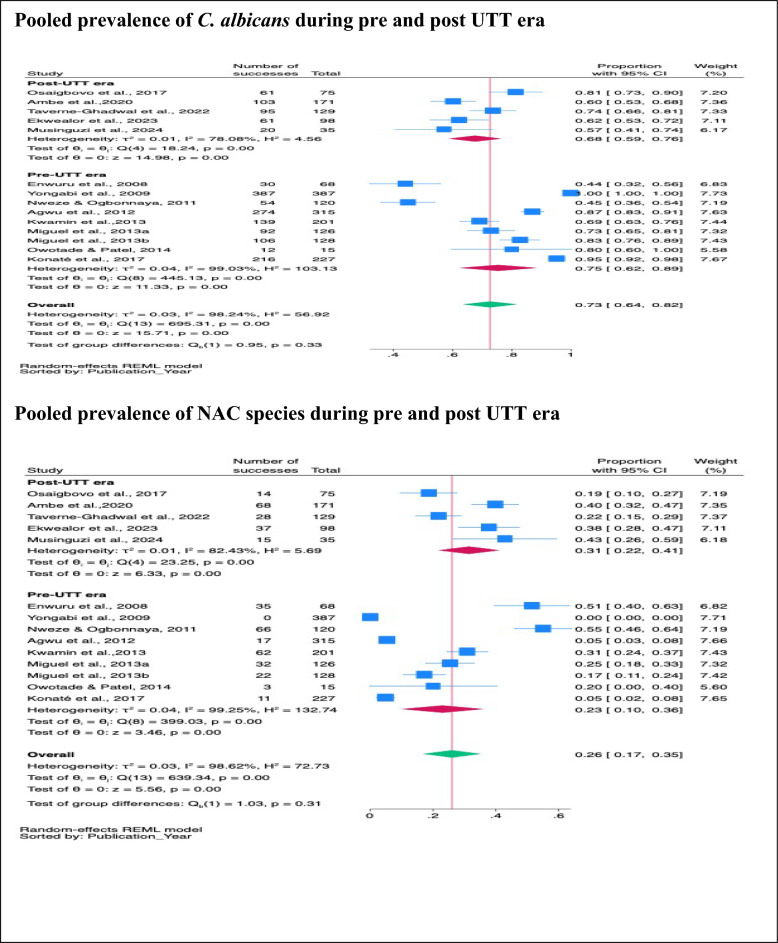
Pooled prevalence of *C. albicans* and NAC species isolated from PLHIV during pre- and post-UTT era

Subgroup analysis revealed no significant difference between the prevalence of *C. albicans* in the post-UTT era (68%, 95% CI 59–76%, *p* < 001) and that of *C. albicans* in the pre-UTT era (75%, 95% CI 62–89%, *p* < 001) (*p* = 0.33). Likewise, the prevalence of NAC species revealed no significant difference in the post-UTT era (31%, 95% CI 22–41%, *p* < 001) and in the pre-UTT era (23%, 95% CI 10–36%, *p* < 001) (*p* = 0.31) (Fig. 4).

#### Prevalence of oropharyngeal and oral candidiasis among PLHIV across different studies in different African countries

The14 studies had a sample size of 4281 participants. Eleven studies reported that the prevalence of oropharyngeal candidiasis ranged from 4.9% in one of the studies in Nigeria to 79.4% on the Ivory coast [11, 12]. The reported prevalence of oral candidiasis was 7.6% in South Africa [33], 48.2% in Chad [14] and 65.3% in Cameroon [30]. Studies conducted in different countries reported varying prevalence rates of oropharyngeal candidiasis. For example, three studies from Cameroon reported rates of 43%, 48%, and 73% [16, 31, 32], 7.6% and 52% in Uganda [15, 34], 79% in South Africa [31], 4.9%, 31.9% and 60% in 3 different studies in Nigeria [27–30], 75.3% in Ghana [35] and 79.4% in the Ivory coast [12] (Table 2).

#### Subgroup country pooled prevalences of oropharyngeal candidiasis and oral candidiasis among PLHIV across different countries in Africa

**Fig. 5:**
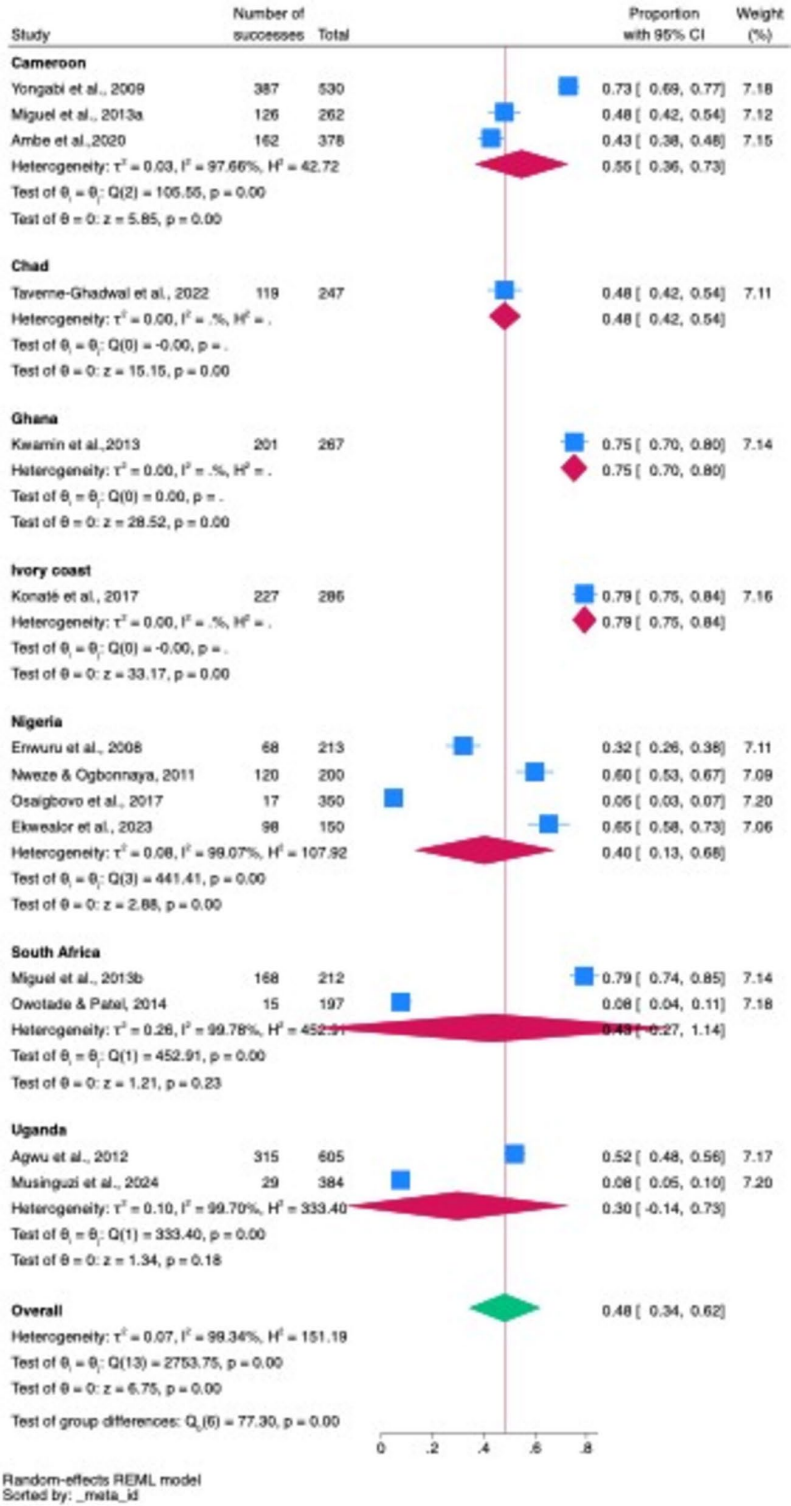
Subgroup pooled prevalence of oropharyngeal and oral candidiasis across different studies in different countries Fig. 6 Regional subgroup pooled prevalence rates of oropharyngeal candidiasis and oral candidiasis in studies in different African regions

After subgroup analysis to determine the pooled prevalence of oropharyngeal and oral candidiasis among PLHIV across various countries, the Ivory Coast presented the highest prevalence at 79%, followed by Ghana at 75%, Cameroon at 55%, Chad at 48%, South Africa at 43%, Nigeria at 40%, and Uganda at 30%. The observed difference in the pooled prevalence across these countries was statistically significant, with a *p* value < 0.001 (Fig. 5).

#### Regional subgroup pooled prevalences of oropharyngeal and oral candidiasis among PLHIV across different regions in Africa

**Fig. 6:**
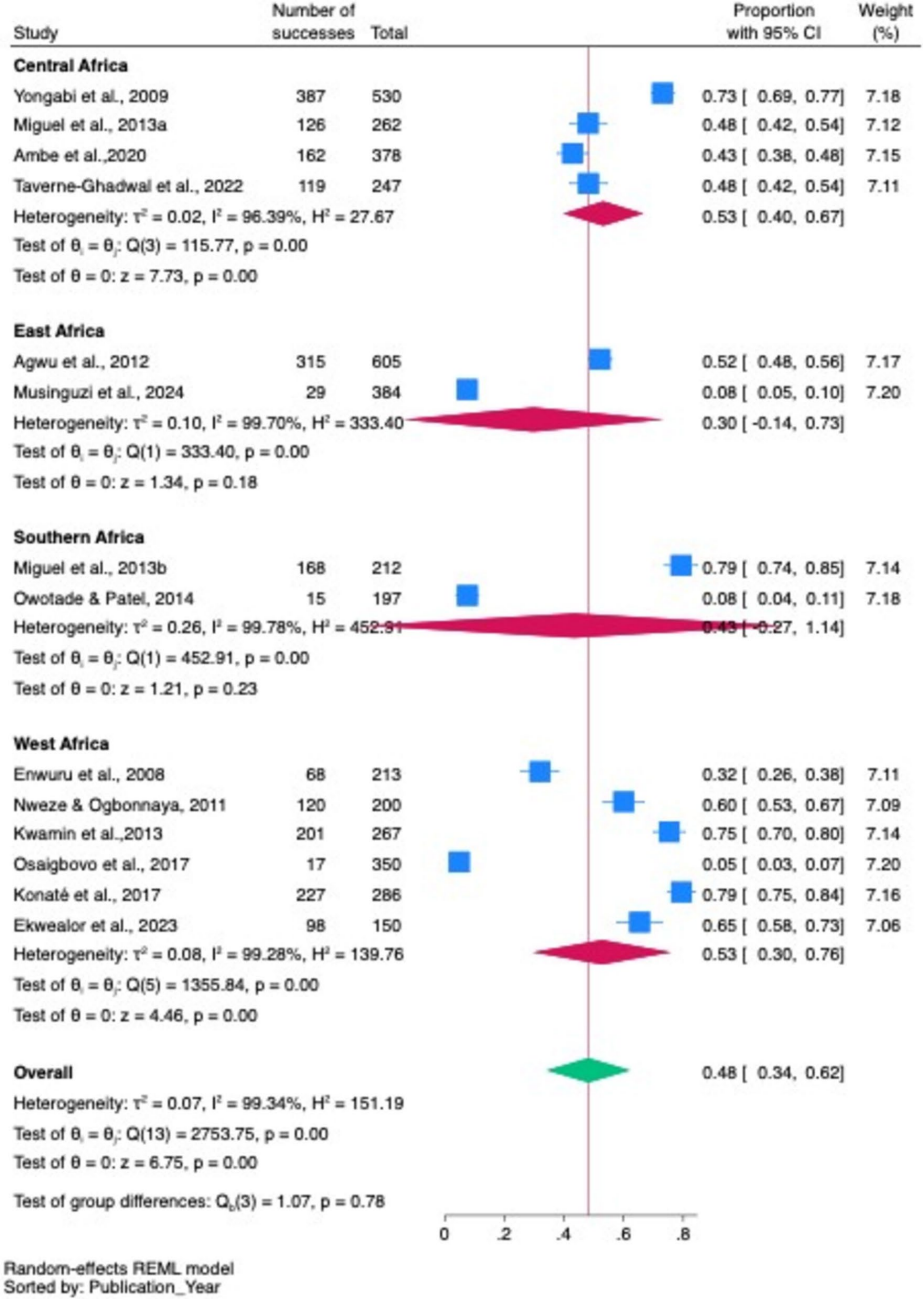
Regional subgroup pooled prevalence rates of oropharyngeal candidiasis and oral candidiasis in studies in different African regions

In regional subgroup analysis, Central and West Africa had the highest prevalence of oropharyngeal candidiasis (53% each), compared to Southern Africa (43%) and East Africa (30%). However, the difference was not statistically significant (*p* = 0.78) (Fig. 6).

#### Overall combined pooled prevalence of oropharyngeal candidiasis and oral candidiasis among PLHIV in Africa

**Fig. 7:**
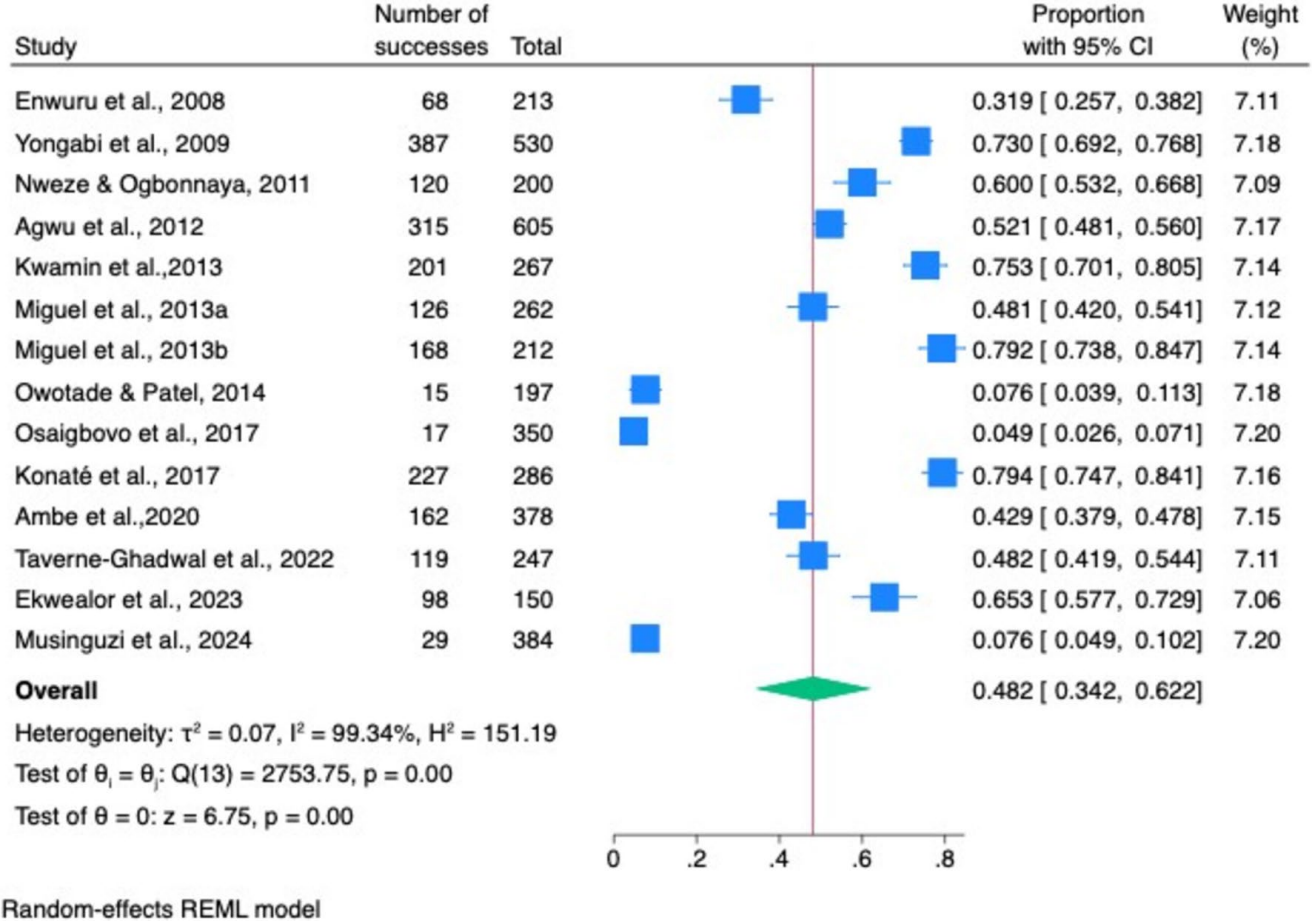
Pooled prevalence of oropharyngeal candidiasis and oral candidiasis in Africa across different studies

The overall combined pooled prevalence of oropharyngeal and oral candidiasis among PLHIV was 48% (95% CI 34–62%) (Fig. 7).

#### Subgroup pooled prevalence of oropharyngeal candidiasis alone and oral candidiasis alone

**Fig. 8:**
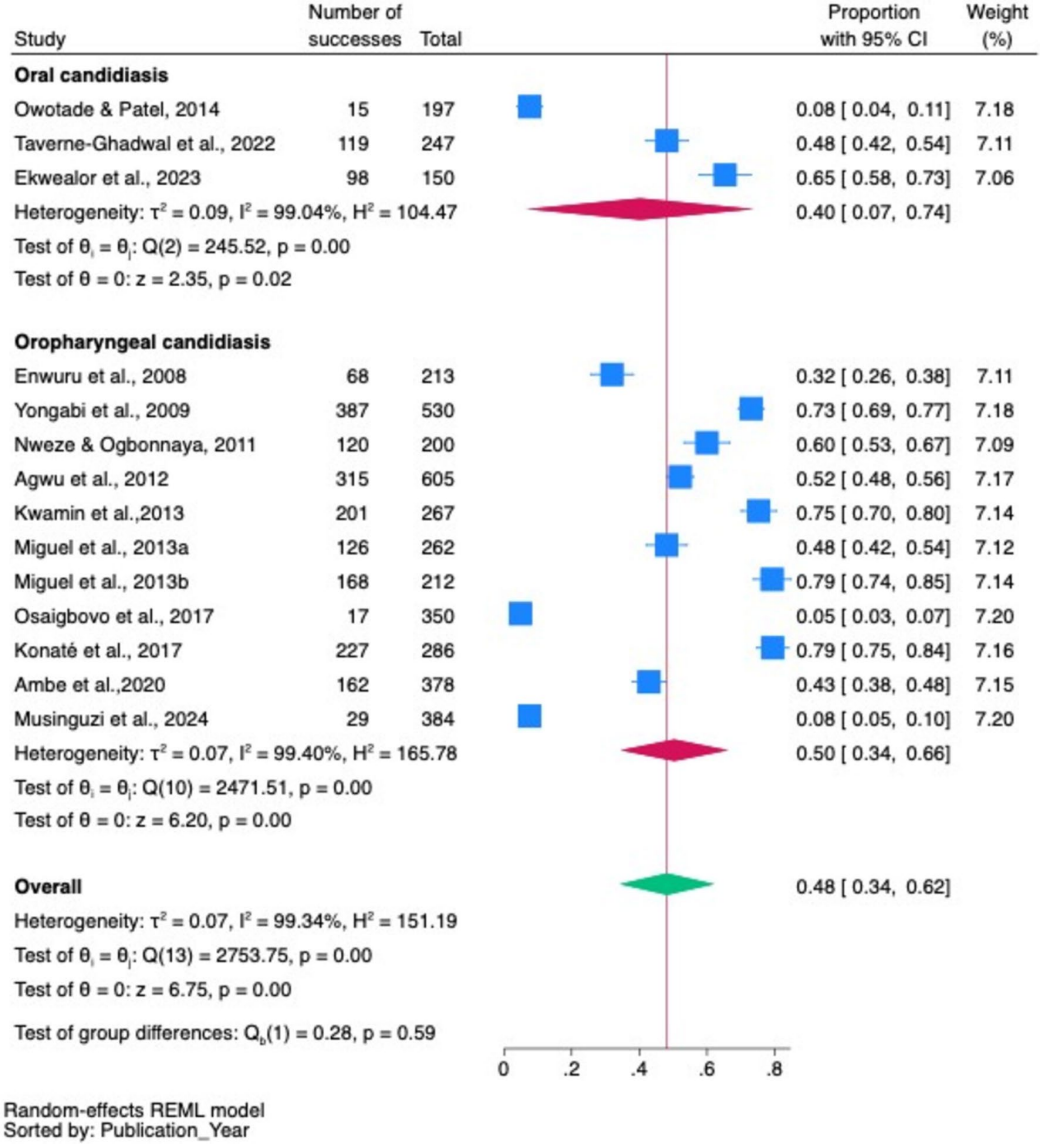
Pooled prevalence of oropharyngeal candidiasis alone and oral candidiasis alone

According to the subgroup analysis, the pooled prevalence of oropharyngeal candidiasis alone was 50% (95% CI 34–66%), whereas that of oral candidiasis alone was 40% (95% CI 7–74%, *p* < 001), but the difference was not statistically significant (*p* = 0.59) (Fig. 8).

#### Pooled prevalence of oropharyngeal candidiasis in the pre- and post-UTT era

**Fig. 9:**
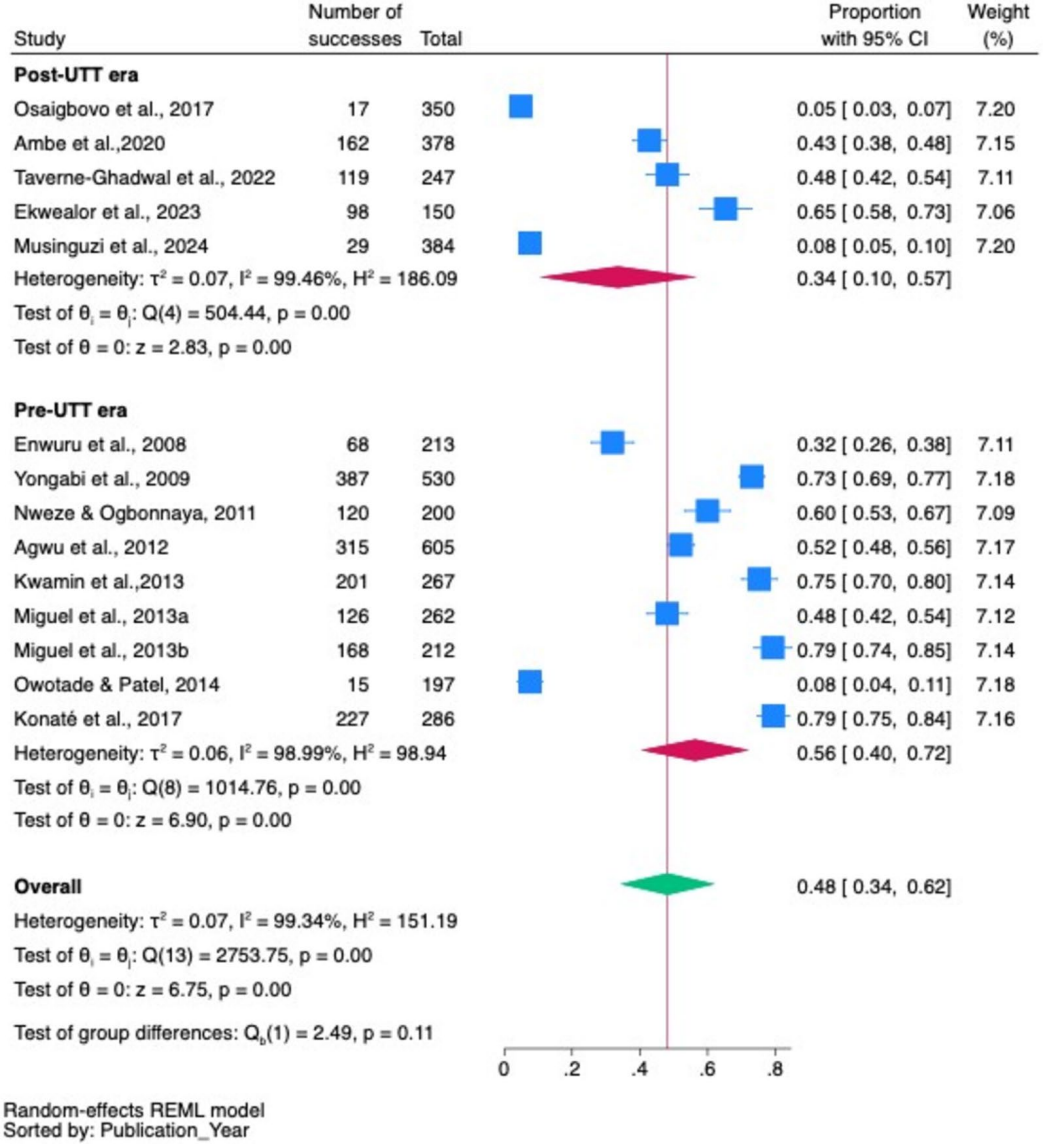
*Pooled prevalence of oropharyngeal candidiasis in* in the pre- and post-UTT era

According to the subgroup analysis, the pooled prevalence of oropharyngeal candidiasis was greater in the pre-UTT era, at 56% (95% CI 40–72%, *p* < 0.001), than in the post-UTT era, at 34% (95% CI 10–67%, *p* < 0.001) (Fig. 9).

#### Trends in the cumulative prevalence of oropharyngeal candidiasis and oral candidiasis

**Fig. 10:**
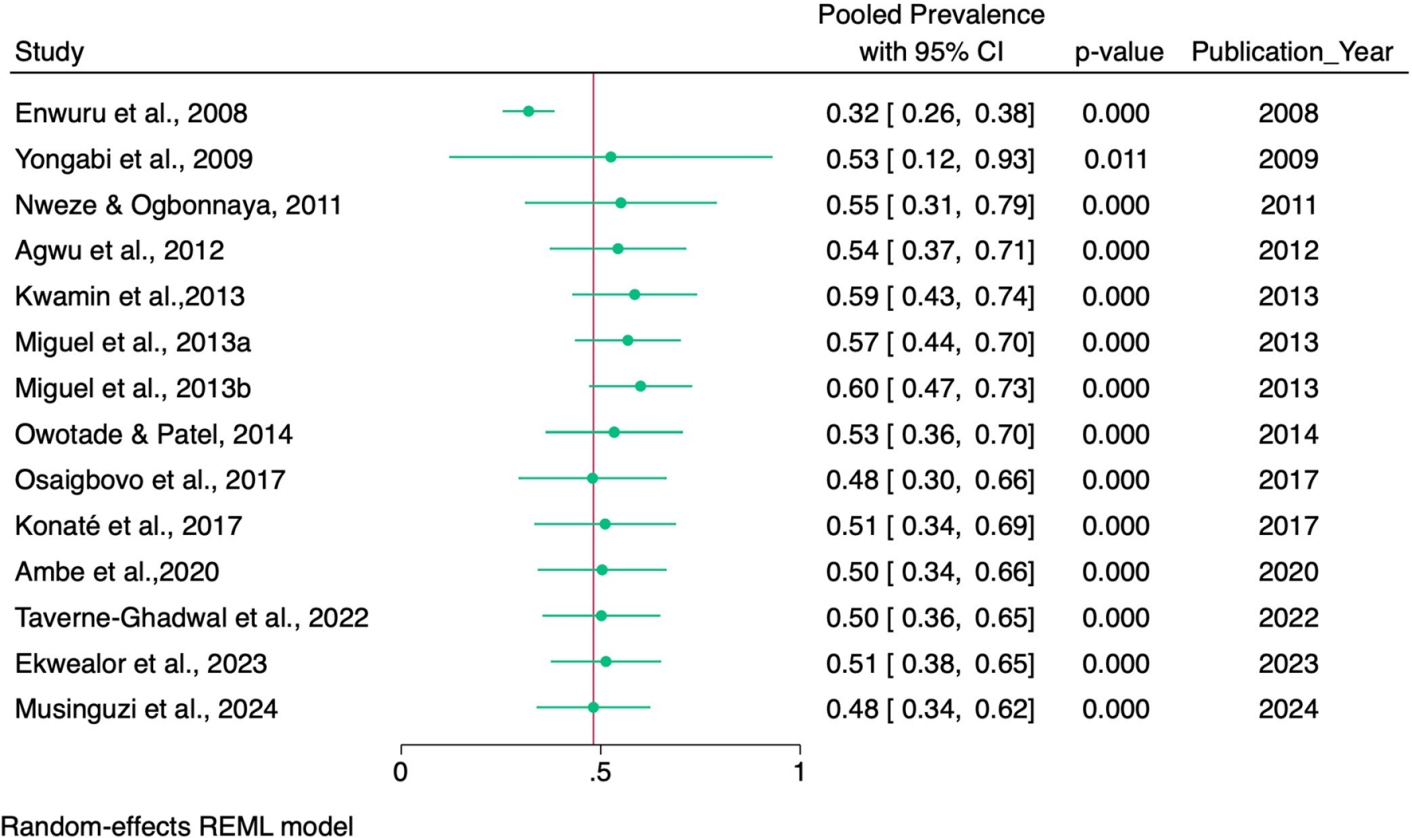
Cumulative prevalence of oropharyngeal oral candidiasis among PLHIV from January 1, 2000, to July 1, 2024

In general, the cumulative prevalence of oropharyngeal and oral candidiasis has declined over two decades. A slight increase was observed from 32% in 2008 to 60% in 2013, and then a decline from 53% in 2014 to 48% in 2017 and 2024 was observed (Fig. 10).

#### Trends in the cumulative prevalence of *C. albicans* and NAC species isolated PLHIV

**Fig. 11:**
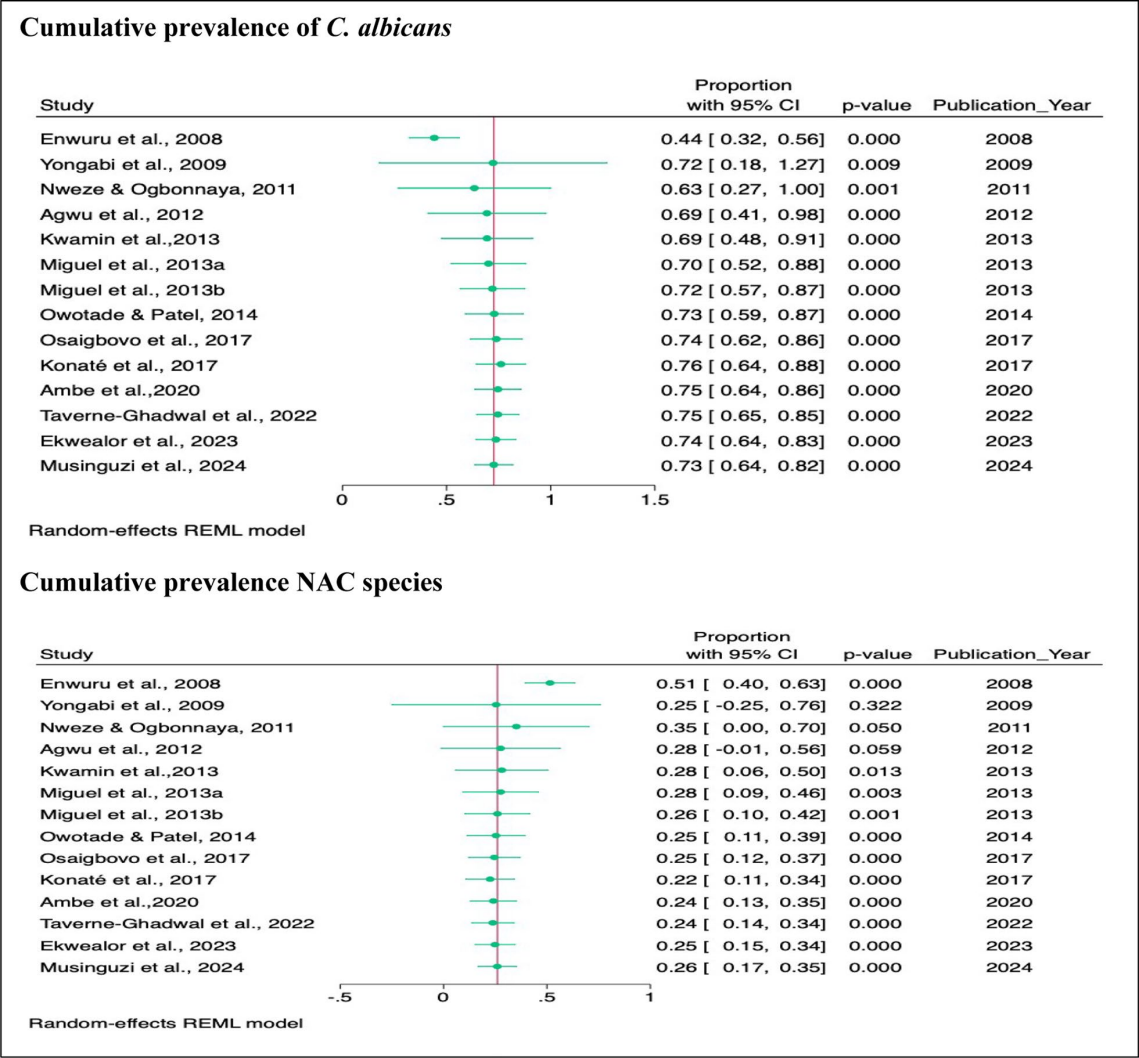
Trends in cumulative prevalence of *C. albicans* and NAC species isolated PLHIV from January 1, 2020 to July 1, 2024

A random effect cumulative meta-analysis was performed to demonstrate the trends. The cumulative prevalence of *C. albicans* increased from 44% in 2008 to 73% in 2014, 76% in 2017, and then declined to 73% in 2024, whereas that of NAC species declined from 51 to 25% in 2014 and then slightly increased to 26% in 2024 (Fig. 11).

#### Risk of *bias* in the included studies

Of the 14 studies, 10 (71.4%) had a low risk of bias, while 4 (28.6%) had a moderate risk of bias (Table 4). In addition, 28.6% of the studies displayed potential bias related to the methods used for identifying *Candida* species.

**Table 4:** Risk of bias assessment of individual studies included in the meta-analysis

Study	Question									Total score	Risk of bias
	Q1	Q2	Q3	Q4	Q5	Q6	Q7	Q8	Q9		
Agwu et al. 2012 [34]	0	0	0	0	0	1	0	1	0	2	Low
Nweze and Ogbonnaya 2011 (Nweze and Ogbonnaya 2011)	0	0	0	0	0	0	0	0	0	0	Low
Osaigbovo et al. 2017 [11]	0	0	0	0	0	0	0	0	0	1	Low
Enwuru et al. 2008 [27]	0	0	0	0	0	1	0	0	0	1	Low
Kwamin et al. 2013 [35]	0	0	0	0	0	0	0	0	0	1	Low
Ambe et al. 2020 [16]	0	0	1	0	0	0	0	0	0	1	Low
Miguel et al. 2013a [31]	0	0	0	1	1	0	1	0	0	3	Moderate
Taverne-Ghadwal et al. 2022 [14]	0	0	1	1	0	1	0	1	0	4	Moderate
Owotade and Patel 2014) [33]	0	0	0	0	0	0	0	0	0	0	Low
Konaté et al. 2017[12]	0	0	0	0	0	0	0	0	0	0	Low
Miguel et al. 2013b [31]	0	0	0	1	1	0	1	0	0	3	Moderate
Ekwealor et al. 2023 [30]	0	0	0	0	0	0	0	0	0	0	LOW
Musinguzi et al. 2024 [15]	0	0	0	0	0	0	0	0	0	0	LOW
Yongabi et al. 2009 [32]	0	0	0	1	1	1	1	0	0	4	Moderate

Key for different questions used to assess the risk of bias

Q1 = Was the sample frame appropriate to address the target population?

Q2 = Were study participants sampled appropriately?

Q3 = Was the sample size adequate?

Q4 = Were the study subjects and the setting described in detail?

Q5 = Was the data analysis conducted with sufficient coverage of the identified sample?

Q6 = Were valid methods used for the identification of *Candida* species?

Q7 = Was the condition measured in a standard, reliable way for all participants?

Q8 = Was there appropriate statistical analysis?

Q9 = Was the response rate adequate, and if not, was the low response rate managed appropriately?

#### Risk of publication *bias*

**Fig. 12:**
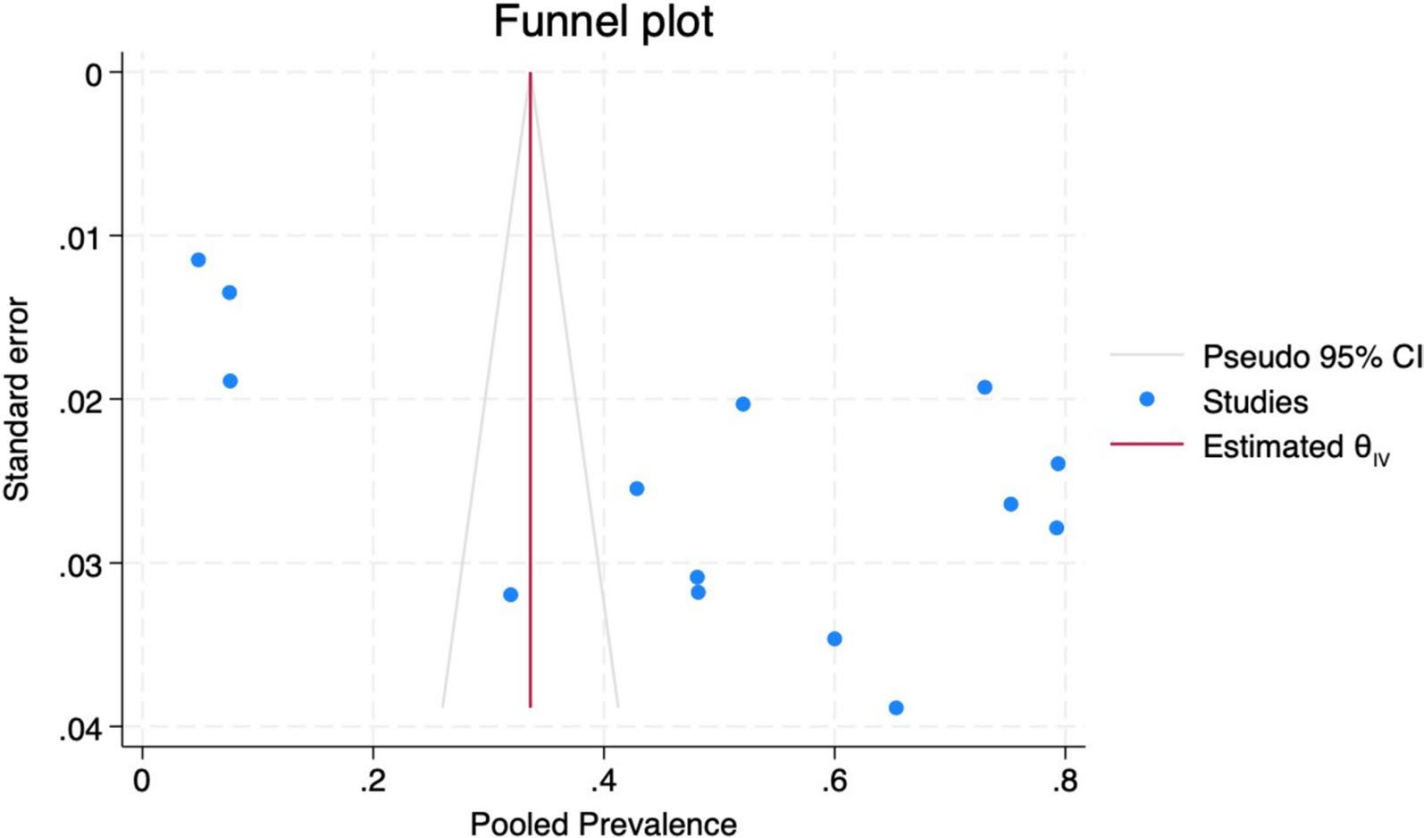
Funnel plot showing the publication bias of the included studies

Publication bias was assessed on the basis of asymmetry of the funnel plot, and statistically, Egger’s test and the trim-and-fill method were applied to correct for possible publication bias at a significance level of < 0.05. According to the funnel plot, it was asymmetrical, the majority of the studies were outside (*n* = 13), and there was significant publication bias both visually (Fig. 12) and via Egger’s test (*p* = 0.029). After a nonparametric trim and fill analysis, imputing to the right and left, one at a time, the prevalence of oropharyngeal and oral candidiasis was still 48%

#### Heterogeneity

There was high heterogeneity across the individual studies reporting the prevalence of oropharyngeal and oral candidiasis (*I*^*2*^, 99.34%, *Q* [13] = 2754, *p* < 0.001**)** (Fig. 7).

#### *Meta*-regression for the overall prevalence of oropharyngeal and oral candidiasis

The results of the meta-regression analysis revealed that the year of data collection, year of publication, UTT era, and African region were not significant sources of heterogeneity for the prevalence of oropharyngeal and oral candidiasis. There were no statistically significant associations between the prevalence of oropharyngeal/oral candidiasis and the year of data collection (coefficient = − 0.016, *p* = 0.198) or the year of publication (coefficient = − 0.013, *p* = 0.364), but there were slight negative trends. The African region revealed no significant associations, with coefficients indicating variability rather than systematic differences. The UTT era analysis suggested a potential trend towards lower prevalence of oropharyngeal and oral candidiasis in the post-UTT era, but this trend was not statistically significance (coefficient = − 0.227, *p* = 0.105) (Table 5).

**Table 5:** Associations between the prevalence of oropharyngeal/oral candidiasis and the year of data collection, year of publication, African region, and UTT era

Variables	Coefficient	*p* value	95% CI
Year data collection	− 0.016	0.198	− 0.041–0.008
Year of publication	− 0.013	0.364	− 0.041–0.015
Post-UTT era	− 0.227	0.105	− 0.502–0.048
East African region	− 0.233	0.350	− 0.722–0.256
Southern African region	− 0.097	0.697	− 0.586–0391
West African region	− 0.003	0.368	− 0.368–0.361

## Discussion

### Principal findings

This systematic review and meta-analysis aimed to determine the distribution of *Candida* species isolated from PLHIV with oropharyngeal and oral candidiasis in Africa. A total of 2,095 *Candida* isolates were reported, of which 78.7% were *C. albicans,* 19.6% were NAC isolates, and 1.7% were not identified specific species level.

In 2095, *Candida* isolates were approximately 32.6% from Cameroon, 17.2% from Nigeria, 16.7% from Uganda, 10.8% from the Ivory coast, 9.6% from Ghana, 6.2% from Chad and 6.8% from South Africa. Despite increasing trends in *C. albicans*, pathogenic NAC species still exist. Hence, there is a need for laboratory diagnosis of oropharyngeal candidiasis and speciation of *Candida* species to improve its diagnosis and management [36].

The combined pooled prevalence of oropharyngeal candidiasis and oral candidiasis was 48%. Subgroup analysis revealed that the prevalence of oropharyngeal candidiasis was 50% and that of oral candidiasis alone was 40%, with a reduction in the prevalence of oropharyngeal candidiasis from 56% in the pre-UTT era to 34% in the post-UTT era.

### Findings in relation to other reviews

Overall, *C. albicans* was the most common species isolated from PLHIV with oropharyngeal and oral candidiasis among PLHIV compared with NAC (21.7%). The high frequency of *C. albicans* was in agreement with other studies that reported *C. albicans* to be the dominant *Candida* species causing oropharyngeal and oral candidiasis in China (71%), Indonesia (50%), India (50%), and Iran (58%) [37–40]. Owing to its stronger ability to adhere to buccal epithelial cells and form complex biofilms, *C. albicans* is isolated more frequently than NAC species that cause oropharyngeal and oral candidiasis [41, 42]. Although the high frequency of *C. albicans* may be a reflection of its virulence, its high prevalence coupled with NAC species could be due to misidentification as a result of the use of less sensitive and specific conventional diagnostic approaches [43].

Our results support observations that have been reported in several other studies identifying *C. albicans* as the most frequent species as well as recognizing the epidemiological existence of NAC species [42, 44]. This has led to the emergence of NAC species as significant *Candida* pathogens. For example, the distribution of NAC species in our study agreed with studies conducted in Indonesia, Iran and India that identified *C. glabrata* (15–19%), *C. krusei* (4.6–15%) and *C. tropicalis* (4.6–10%) as the most prevalent NAC species [38–40]. The emergence of NAC species may be due to the use of antimicrobial agents, such as antifungals, antiretrovirals, and antibiotics [23]. Exposure to these agents may exert positive selection pressure on NAC species, which are considered intrinsically resistant to antifungal agents [45, 46]. In addition, recent studies have demonstrated that *C. albicans* and *C. glabrata* have a synergistic relationship in which *C. albicans* facilitates the initial development of oropharyngeal candidiasis infection by *C. glabrata* [42, 47]*.*

Uganda reported the highest percentage of unidentified *Candida* species. This is unsurprising, as accurate identification of *Candida* species has been recognized as a challenge in Uganda, and improved laboratory techniques are needed to enhance the definitive diagnosis of candidiasis [48, 49].

Generally, we noted high variability in the prevalence of oropharyngeal and oral candidiasis among PLHIV across different studies and countries, ranging from 4.9 to 79.4% in different studies [11, 12], with pooled prevalence of 30% in Uganda and 79% in Ghana. Differences in ART access, UTT policy implementation, and treatment adherence likely influenced CD4 counts, affecting oropharyngeal candidiasis rates across studies and countries. In addition, varying use of prophylactic drugs like fluconazole could impact infection rates, while higher rates of co-infections and NCDs, such as tuberculosis and oropharyngeal cancer, may further contribute to differences in the prevalence.

In addition, Central and West Africa had high prevalence compared to Southern Africa and East Africa. However, the meta-regression results revealed that none of the regions had a significant association with the prevalence of oropharyngeal and oral candidiasis. This suggests that the prevalence may not vary significantly across different regions of Africa or that any observed differences could be due to random variation rather than systematic differences.

Our obtained overall combined pooled prevalence of oropharyngeal and oral candidiasis of 48.0% in Africa was higher compared to 29% in Europe, 30% in America, and 39% in Asia [50]. Differences in immune status, diagnostic approaches, CD4 levels, availability of ART, treatment of candidiasis and geographic location have been outlined as possible reasons for differences in the prevalence of oropharyngeal and oral candidiasis [51]. Socioeconomic factors such as poverty, education level, and limited healthcare access likely contributed to the higher prevalence of oropharyngeal and oral candidiasis in Africa, where poverty rates are higher, and healthcare access is more limited. In addition, behavioral factors like poor oral hygiene, smoking, alcohol consumption, and illicit drug use, which vary across populations, may have increased the risk of infection. The high burden of HIV/AIDS in sub-Saharan Africa also heightens the risk of candidiasis caused by various *Candida* species.

Our findings align with other reviews highlighting oropharyngeal and oral candidiasis as a persistent challenge among PLHIV in Africa [50, 52].

The pooled prevalence of oropharyngeal candidiasis was generally lower in the post-UTT era compared pre-UTT, as evidenced by a decline in the cumulative prevalence. This can be attributed to the widespread availability of ART and the implementation of UTT policies in many African countries during the data collection period (2017–2023) for studies in the post-UTT era. This affirms increasing evidence that oropharyngeal candidiasis among PLHIV is declining as previously reported in other studies [23, 53, 54]. Immediate ART initiation for all individuals diagnosed with HIV, regardless of CD4 count or clinical stage, along with good adherence to treatment, likely improved CD4 counts and immune status in PLHIV during the post-UTT era, reducing the incidence of opportunistic oropharyngeal and oral candidiasis. Our findings are consistent with previous studies reporting a decline in oropharyngeal candidiasis following the introduction of ART and the implementation of UTT policy [20, 21]. Accurate laboratory diagnosis of oropharyngeal and oral candidiasis as well as precise speciation of *Candida* species are still crucial for improving both diagnosis and management.

### Implications of this review for health professionals, future research, and policy

Given the occurrence of oropharyngeal and oral candidiasis caused by both *C. albicans* and NAC among PLHIV in Africa, it is essential that clinicians, laboratory professionals, and microbiologists adopt accurate molecular diagnostic approaches. These approaches should be used to differentiate *Candida* species and determine their antifungal susceptibility profiles. Doing so could continuously help reduce the prevalence of oropharyngeal and oral candidiasis and enhance the quality of life for PLHIV.

Although both pathogenic *C. albicans* and NAC species were reported in this review, we did not examine the virulence attributes and antifungal resistance patterns of *Candida* species. Understanding virulence factors is vital for understanding oropharyngeal candidiasis pathogenesis and consequently helps improve the diagnosis and therapeutic treatment of oropharyngeal and oral candidiasis among PLHIV. This area can be strengthened in future studies. Policy makers and actors should consider investing in strengthening mycology laboratories and supporting research efforts focused on *Candida* species, antifungal resistance, and related diagnostic innovations.

### Strengths and limitations of this review and *meta*-analysis study

The strengths of this review and meta-analysis study were the use of a rigorous search of the PubMed, Scopus, and EMBASE databases following the PRISMA statement and the inclusion of oropharyngeal and oral candidiasis cases confirmed by microbiological laboratory methods. We were able to evaluate the impact of the UTT policy on oropharyngeal candidiasis and oral candidiasis. However, this meta-analysis had the following limitations. The studies included in this review had a wider range of oropharyngeal candidiasis prevalence (4.9–79.4%), and there was high publication bias, as reflected by heterogeneity. This could have been due to differences in the immune status of the studied population. However, we addressed publication bias by performing subgroup analysis, meta-regression, and cumulative prevalence analysis.

We considered the study period (2000–2024) and excluded studies that did not include speciation for *Candida* species. In addition, our research team had no expert who was well conversant in languages other than English; thus, we considered papers that were written in English. All these limitations could have introduced bias in the overall pooled prevalence of oropharyngeal/oral candidiasis among PLHIV in Africa.

## Conclusion

While *C. albicans* remain, the predominant species causing oropharyngeal and oral candidiasis among PLHIV in Africa, NAC species also contribute significantly to the infection burden. Despite ART and UTT policy, candidiasis remains prevalent, emphasizing the need for targeted interventions.

## Data Availability

The datasets analysed during the current study are available from the corresponding author upon reasonable request.

## Abbreviations

ART: Antiretroviral therapy
PLHIV: People living with human immunodeficiency virus
UTT: Universal test and treat
NAC: Non-*albicans Candida*
PRISMA: Preferred reporting items for systematic review and meta-analysis
